# Medical Items

**Published:** 1907-11

**Authors:** 


### MEDICAL ITEMS.

### SYPHILO-DERMO-UROLOGIC MAXIMS.

It is not necessary that a mole be elevated above the surface of the skin; it often manifests itself as a dark brown or black stain.

It is a good rule to have all ointments made extemporaneously, as they can be better adapted to the case and the ingredients be fresh.

Chloasma, mother's mark, or moth is an essential pigmentation of the skin, and not due to liver trouble. It is absurd to bother the liver to cure it.

---

Itching is not a symptom limited to scabies, lice, and eczema. There are many inflammatory as well as parasitic diseases in which itching is a prominent symptom.

---

In order to make hair, horny cells, or animal parasites translucent it will be found that a preliminary soaking of them in the dilute liquor potassa will accomplish the purpose.

---

When the pubis and neighborhood itch look for animal parasites. The writer has seen a patient treated for eczema when the cause was the pediculosis pubis.

---

Do not lay much store upon the various so-called dyscransiae. Very often the solution of the dyscrasia lies in the proper use of a liberal amount of soap and water.

A little more time and milder measures will eventually bring more satisfactory results, and a safer termination in gonorrhoea.

---

Lupus vulgaris is also due to the tubercle bacillus, and reqnires strenuous measures in its treatment. The Roetgen-ray treatment will bring about rapid cicatrization and, later on, carcinoma is apt to develop in the scar.

---

It is not absolutely necessary to cauterize every chancroid.

---

Herpes preputialis is a trouble which is not easy to bring to a successful issue because it is so prone to relapses at varying intervals.

---

A great popular fallacy which should be eradicated from the minds of the laity is that menstrual blood or leucorrhoea will give rise to gonorrhoea.

The so-called "quick-cure" of gonorrhoea is the most dangerous practice known, as it is very liable to lead to the development of stricture.

Forcible divulsion of a stricture is both barbarous and inefficient. Gradual dilatation is more satisfactory to the patient but does not result in good that is permanent.

A mistake which is easily made is to take a chancre redux for a second syphilitic infection. The cases of second infection are sufficiently rare to make the wish the father of the thought.

The pus of a gumma which is breaking down is infectious and too much confidence must not be placed in the idea that the lesions of tertiary syphilis do not infect.

It is not a safe method to depend entirely upon the fact that the chancroid is a bacilliary disease and depend upon antiseptics too much. This lesion may become phagedenic and be very destructive.

Too much confidence must not be placed in the iodides as curative means in syphilitic processes. It very often happens that they are more irritating than curative and cannot always be depended upon.

In lesions of the motor nerves as evidenced by paralysis of one of the limbs, and such as occur later in syphilis, the iodides often fail. It is in these cases that good results are found to follow the hypodermic injections of soluble mercurials.

Care should always be taken not to needlessly alarm a patient. Gain his confidence and he will come oftener and depart more satisfied.

Too much meddlesomeness in the treatment of veneral diseases is as harmful as in obstetrics.

A good rule of practice to pursue is to employ methods that are cleanly and withal efficient, and in nothing does this apply more than in venebeology.

A lesion which requires care and attention in formulating a diagnosis is the chancre, more especially when located on the tonsil, in the rectum or on the cervix uteri.

—Extracted from the American Journal of Dermotology.

---

At the recent meeting of the Southern Medical Association in Birmingham the following officers were elected: President, Dr. B. L. Wyman, of Birmingham; Vice-presidents, Dr. W. P. McAdory, Alabama; Dr. H. M. Folkes, Mississippi; Dr. Frank Watson, Louisiana; Dr. J. R. Holden, Florida; Dr. Raymond Wallace, Tennessee, and Dr. A. L. Fowler, Georgia.

Dr. Oscar Dowling, of Shreveport, La., was elected secretary-treasurer.

The following sectional officers were chosen:

Dr. Seale Harris, Mobile, president, and Dr. H. E. Mitchell, Birmingham, secretary, medicine; Dr. W. F. Westmoreland, Atlanta, president, and Dr. Jere L. Crook, Jackson, Tenn., secretary, surgery; Dr. J. F. Herron, Jackson, Tenn., president, and Dr. A. B. Harris, Birmingham, secretary, ophthalmology. The appointment of Dr. W. W. Butterworth as councilor for Louisiana was announced by President Wyman. A councilor for Florida will be appointed later. The others hold over.

Atlanta was chosen as the place for next meeting, which will be held on the first Tuesday in November next year.

---

### EIGHTH ANNUAL MEETING OF THE NATIONAL ASSOCIATION FOR THE STUDY OF EPILEPSY AND THE CARE AND TREATMENT OF EPILEPTICS.

The eighth annual meeting of the National Association for the Study of Epilepsy and the Care and Treatment of Epileptics was held at the Jefferson Hotel, Richmond, Va., October 24th-25th.

Addresses were made at the first session by Hon. Claude A. Swanson, Governor of Virginia; Hon. Carlton McCarthy, Mayor of Richmond; Dr. Paul B. Barringer, President of the Medical Society of Virginia, and Dr. Ennion G. Williams, President of the Richmond Academy of Medicine. Responses to these were made by Dr. Everett Flood, President of the Association, and Dr. Wm. Spratling, of the Craig Colony for Epileptics, Sonyea, N. Y.

Reports from all States in this country which are now caring for epileptics were made by the Secretary.

The program of the papers is as follows:

Dr. H. M. Weeks, Skillman, N. J.—"The Utilization of Epileptic Labor."

Rev. J. Duncan MacNair, resident Chaplain of the Craig Colony for Epileptics at Sonyea, N. Y.—"Colony Life of an Epileptic—Social and Religious."

Dr. James F. Munson, Sonyea, N. Y.—"Sewerage Disposal—the Construction and Work done by the Filter Beds at Sonyea, N. Y."

Dr. J. S. DeJarnette, Staunton, Va.—"Epilepsy: Its Definition, Treatment, etc.

Dr. Thomas C. Fitzsimmons, Wilkesbarre, Penn.—"Alcohol as a Primary and Exciting Cause of Epilepsy."

Dr. M. B. Hodskins, Palmer, Mass.—"The Etiology of Epilepsy."

Dr. William P. Spratling, Sonyea, N. Y.—"The Systematic Treatment of Epilepsy versus Its Treatment by an Occasional Consultation and Prescription."

Dr. J. Allison Hodges, Richmond, Va.—"The Value of Elimination in the Treatment of Epilepsy."

Dr. William T. Shanahan, Sonyea, N. Y.—"Pulmonary Oedema as a Complication of Epileptic Seizures."

Dr. Matthew Woods, Philadelphia, Pa.—"Surgery as a Therapeutic Measure in the Cure of Epilepsy."

Dr. H. H. Levy, Richmond, Va.—(Title to be announced later.)

Dr. Stuart McGuire, Richmond, Va.—"Surgical Intervention in the Treatment of Epilepsy."

Dr. A. V. Cooper, Palmer, Mass.—"Injuries to Epileptics."

Dr. Edward A. Kennedy, Palmer, Mass.—"Myoclomus Epilepsy."

Dh. L. Pierce Clark, New York City.—"Cranial Nerve Fits."

Dr. D. D. Wilcox, Petersburg, Va.—"The Relation of Eye Defects to Epilepsy."

Dr. G. Kirby Collier, Sonyea, N. Y.—"Some Features of an Epileptic Aura."

Dr. James E. Munson, Sonyea, N. Y.—"The Heart's Action Preceding the Seizure."

### ANTIPHLOGISTINE VERSUS OPIUM.

Inflamed states of the various organs of the body frequently give rise to pain of such urgent character as to demand active steps looking to its relief. Upon seeing the patient for the first time (he has called his physician because his suffering has become intolerable) the medical attendant is met with a peremptory demand for relief from the suffering.

With a willingness, which frequently overrides their better judgment, some physicians resort to the hypodermic needle indiscriminately, and, in too many cases, a greater evil has followed the lesser one. The free habit of using morphine or some other form of opium is not a judicius practice, and for several reasons. The exact seat of an inflammation, for instance, might become difficult to locate, and thus a clear diagnosis interfered with. But the greater objection to the use of opium is the possibility of adding a recruit to the ever growing army of habitues.

Every time there occurs to a doctor the apparent need for opium he should deliberate well before resort is had to the needle. If, after careful deliberation, his best judgment advises the use of opium, it should be given in some form by mouth. If the needle is used the patient at once knows what he is getting, but he is not likely to acquire this information if it be given otherwise.

For relieving the pain of the inflammations Antiphlogistine will easily take the place of opium. The relief may not be so prompt and so complete, but the edge of the suffering is taken off within a short time, and soon the patient is in a comfortable condition and has escaped the possibility of becoming addicted to the drug. There is not the likelihood that the patient, relieved from pain by it, will begin eating or using Antiphlogistine in any other way which likelihood is the greatest disadvantage of opium.

In the future let your morphine become stale, and keep your Antiphlogestine fresh—use it in inflammation.—The Medical Era.

### THE CARE OF GROWING GIRLS.

One of the most responsible tasks of the family physician is to advise parents of girls entering upon their 'teens, as to the diet, mode of life, and hygienic measures best calculated to preserve the health of budding womanhood. In dealing with these cases the practitioner is often called upon to treat the anemia which in such a large proportion of instances characterizes the unfolding of the growing girl. Full well does the family doctor grasp the meaning of this anemia, and the vast importance of combating it before it is too late,—before the impoverished condition of the blood of puberty has left its imprint upon the powers of resistance of the adult organism; has done permanent damage to the future woman and the future mother.

Unsuitable diet, an overindulgence in sweets or spices, over-study, lack of fresh air, and physical exercise, indulgence in late hours, and abandonment to novel reading, to tight lacing, and other abominations of dress, contribute their quota to the causes of anemia in the growing girl. Each of these factors is, of course, removable by good common-sense advice to parents and by proper exercise of discipline. Still, when the damage has been done, we must assist nature in its generous work of restoration, and here it is that we are obliged to give that sovereign cure of impoverished blood, iron, in such form as may best be suited to these cases.

The question as to what form of iron we should give to produce the best possible effects has been solved by both experimental and clinical researches conducted during the past twenty-five years—ever since Bunge and Hamburger experimentally demonstrated the inferiority of inorganic preparations (Morat and Doyen, Traite de Physilogie, Paris, Masson 1904, I, 467). Iron, in the anemia of puberty, produces the best effects when given in a form that will stimulate digestion and increase assimilation, i. e., in the form of the peptonate. With it should always be combined that second hematinic and the two are best given in the form of the well-known solution which has been shown to enhance the value of iron,—manganese,—styled "Pepto-Mangan' (Gude.)

With this may be given, in the anemia of growing girls, minute doses of Fowlers' Solution, or else equally small doses of strychnia which may be incorporated with Pepto-Mangan as indicated in individual cases.

Pepto-Mangan has a great advantage over other forms of iron medication in that it does not constipate. Girls at puberty, however, are notoriously prone to constipation. Therefore, this should receive proper attention, chiefly in the regulation of diet, including a sufficient amount of fruit, raw and cooked, and of cereals giving a large residue of cellulose.

With this method of treatment many a physician has achieved success which was rewarded tenfold, by the sight of rosy faces and bright eyes.

---

### THE PATHOLOGY AND TREATMENT OF HAY FEVER.

---

One of the most striking pathological features of this malady is a turgescence of the turbinal tissues due to extensive dilatation of the capillaries. That this is the result of an angioneurosis, involving a more or less pronounced vaso-motor paralysis, is pretty generally conceded.

In the treatment of hay fever with Adrenalin Chloride it has been suggested that weak solutions, frequently applied, are apt to yield better results than the occasional application of strong solutions. The application of the solution of Adrenalin Chloride stimulates the vaso-motor supply, resulting in a contraction of the capillaries. Overstimulation, by reaction, is very sure to result in the complete other hand, gentle stimulation with weak solutions is not very likely to be followed by a reaction.

Solution Adrenalin Chloride (1-1000) may be diluted with normal salt solution and sprayed into the nares and pharynx.
paralysis of the vaso-motor supply in the region affected. On the
Adrenalin Ointment may be applied to the turgescent nasal muco-
obvious reasons. This product contains one part of Adrenalin Chloride in one thousand parts of an aromatized oil base with 3 percent Chlorotone. It is vaporized by means of a nebulizer.

Adrenalin Ointment may be applied to the turgescent nasal mucosa by means of a cotton applicator. Henry Guy Carleton (Therapeutic Gazette June, 1907) says that "Relief can be accomplished more quickly by smearing one or two minims of ointment containing 1:1000 of Adrenalin between the brows and half-way down the side of the nose than by the inunction and spraying of the nasal mucosa." The modus operandi is explained as follows:

"The effect is to delay the irritation of the supraorbital, supratrochlear, and infratrochlear and frontal nerves, and the superior and inferior nasal, and nasal rami of the superior maxillary, and the naso palatine nerves, all of which are involved in a severe attack. Those rami in the posterior rares which may be affected will be relieved simultaneously, exactly as all branches of the supraorbital affected in a supraorbital neuralgia are relieved when an application of Adrenalin Ointment is applied only to the supraorbital foramen."

Messrs. Parke, Davis & Co. issue a brochure on the treatment of hay fever which will be sent to any medical man upon request. We suggest to our readers that they send for the brochure, as hay fever is an exceedingly interesting and timely subject.

### ASTHMA

The doctor who treats all cases of Asthma alike as a separate and distinct disease is making a big mistake. At best it is but a symptom and may be due to a variety of causes. A common cause is tbe gouty diathesis, or the toxaemia of faulty metobolism constituting gouty or toxic asthma. The best treatment for asthma is that which removes the cause in each case. To overcome gouty asthma requires only to correet the gouty diathesis

For this purpose there is no better remedy than Alkalithia. In many cases its action is almost magical, but, it is not a "cure-all" for asthma. Try it in gouty, or toxic asthma, only, and it will not disappoint.

---

Dr. Stephen L. Strickler, of Boggstown, Indiana favorably comments on teh action of Cactina Pillets as follows:

"I have used Cactina Pillets for ten years and can say they are more to be relied on than most anything in medicine that I know. They surely must be made of the drug gathered at the most favorable time of the year, because the Cactus you buy on the market is not reliable." Cactina Pillets have been on the market for twenty years and the testimony of this kind has been heaped upon it by the medical profession. It is being employed with benefit in functional, cardiac and circulatory disturbances and exhibits no cumulative action.

---

### H-M-C- AND A HAPPY DELIVERY.

A few days ago I was called to see a case of obstetrics. The lady was a primipara, twenty-four years old, was anemic, dropsical, with a very bad heart. She began having pains on Sunday afternoon, and I was called Monday morning. She was having pains at intervals of five minutes, but the os did not dilitate. During the day and up to ten oclock the pains grew stronger, were very severe, but with little dilitation of os, patient almost exhausted. I gave 1-2 size H-M-C (Abbot) at 10 p. m. She was sleeping thirty minutes after and was delivered of a fine boy at 2 a. m. Complained some during last three or four pains. I was delighted and so was the patient.

J. H. HAMMOND,

Enigma, Ga.

W. S. WATSON. J. A. PICKARD

WATSON & PICKARD

Proprietors THE GRAND PHARMACY

PEACHPREE ST., ATLANTA, GA.

PHYSICIANS' SUPPLIES CLINICAL THERMOMETERS

PRESCRIPTIONS

HOSPITAL SUPPLIES SURGICAL DRESSINGS

Many a man tries to run a forty horse-power automobile on a five horse-power salary.

---

The cannibals would probably appreciate our missionaries more if we sent them canned.

---

The average man spends most of his time between plans for the future and regrets for the past.

—selected

---

Many children are so crammed with everything that they really know nothing.

In proof of this, read these veritable specimens of definitions, written by public-school children:

"Stability is taking care of a stable."

"A mosquito is the child of black and white parents."

"Monastery is the place for monsters."

"Tocsin is something to do with getting drunk."

"Expostulation is to have the smallpox."

"Cannibal is two brothers who killed each other in the Bible."

"Anatomy is the human body, which consists of three parts, the head, the chist, and the stummick. The head contains the eyes and brains, if any. The chist contains the lungs and a piece of the liver. The stummick is devoted to the bowels, of which there are five, a, e, i, o, u, and sometimes w and y."—Everybody's Magazine, Nov., 1907.

### DR. PETTEY'S EASTERN RETREAT.

Dr. Geo. E. Pettey of Memphis, Tenn., has associated himself with Dr. Wm. F. Ridgway of Atlantic City, N.J., and they have opened a Retreat at 3303 Pacific Ave., Atlantic City for the treatment of alcohol and Drug Afflictions by the methods introduced to the profession by Dr. Pettey. In announcing the opening of this eastern branch of his work, Dr. Pettey ays that some years ago he had Drug Habitues in his own clientele for whom he was unable to secure relief by any of the methods of treatment then in vogue. This led him to undertake a thorough study of the Narcotic Drug Addictions. Two years spent in that work, he says, enebled hi mto form definite conclusions as to Pathoogy of these addictions. With that knowledge to guide him, a rational course of treatment was devised, every detail of which was published to the profession, See Therapeutic Gazette, Oct 1901., and subsequent articles.

He says is was his hope that reputable institutions engaged in the treatment of this unfortunate class of sufferers would avail themselves of the benefits of his study of this subject, but thus far few of them have done so. The majority seem to be still "Wedded to their Idol," the Gradual Reduction Method. In Dr. Pettey's judgment that method of treatment does not give this class of patients the relief to which they are entitled. Since others do not seem to be inclined to acknowledg ethe advantages of Dr. Pettey's method and to give their patients the benefits of it, he is undertaking to meet the demand for such a treatment in the eastern states by opening the Atlantic City Retreat.

Dr. Wm. F. Ridgway, who has assumed the Madical Directorship of the new institution is a graduate of Jefferson Medical College and holds positions of honor in the profession of his city. This, with Dr. Pettey's established reputation is a sufficient guarantee that the new institution will be conducted according to the most scientific principles and upon the highest ethical lines.

Those wishing further information should address The Dr. Pettey Retreat, 3303 Pacific Ave., Atlantic City, N. J. ,or Dr. Geo. E. Pettey, Memphis, Tenn.

Authorities—and our experience teaches that the most important step toward the amelioration of Chronic Nasal Catarrh is cleanliness. A generous use of an alkaline antiseptic with spray sufficiently stimulating to encourage the formation of new blood vessels and nvigorating those that have remained. GERMILETUM has proven in my hands the ideal solvent and alkaline antiseptic, a dnwill effectually cure the most advanced cases of Chronic Nasa Catarrh.

The average income of the physicians of Berlin is said to be more than $2,000 per annum.

### REFORMERS.

Railroads, trusts and all industries
May be mighty full o' sin;
But, b' gosh, it's only through 'em
That a poor man hez "look't in."

"Bust the Trusts" is therefore foolish,"
As a cure for all yer ills;
An' the chap wot shouts it loudest
Ain't the chap wot pays the bills.

This old land ain't goin' ter pieces—
Few would change it if they could;
An' there's lots of things wuth prais'n',
If ye'r' lookin' for the good.

So, old man, there's nothin' ails yer
But a case o' bein' workt,
By a lot o' slick "reformers"
Who their own backyards hev shirkt!

Chuck it all and lets go fishin'—
Wot's the use o' feelin' sad,
Just because a few old croakers,
Tell ye that ye'r' rott'n bad?

—Diogenes Tub, Esquire, M.D.
Cross Roads, U. S. A.

### OUT OF THE SCHOOL-ROOM.

A school-boy was asked to give some information in regard to the Cary sisters, the once famous New England poets, and he said of them:

"The Cary sisters were two poets who lived in Massachusetts most of the time. They went to New York where they made many fast friends. Their fastest friend was John G. Whittier."—November Lippincott's.

### A NEW MYSTERY STORY—"UNDER THE BLACK CASSOCK."

One of the best mystery stories we have read in a long time is Edith Morgan Willett's novel, "Under the Black Cassock," which is published complete in the November Lippincott's. The opening scenes are laid in New York, after which the action shifts to Charleston, and the characters are society folk of the intelligent, well bred kind which really exist, and not at all the hysteric, exotic, little-short-of-idiotic sort that are pictured so often in the so-called society stories. The plot is quite unexpected. Among the shorter fiction is a charming story called "The First Hurdle," by John Reed Scott, who won recognition with his two novels, "The Colonel of the Red Huzzars" and "Beatrix of Clare." "The Blood o' Innocence," by George L. Knapp, is a strongly dramatic story of a feud in England in the days when men wore swords. "The Old Folks at Home," by Sarah Chichester Page, is a delightful sketch of Virginia life. Other note-worthy contributions in the fiction line are by Mabel Nelson Thurston, George Brydges Rodney, Joseph M. Rogers, and Mrs. E. Ayrton-Zangwill. There is another of Mrs. John van Vorst's excellent Parisian articles, this time on "French School-Girls of To-day." Other papers are "A Canadian Heronry," by Bonnycastle Dale; "The Brutality of the Matinee Girl," by Juliet Wilbor Tompkins; "The Vehicle of Disease," by Lewis B. Ely; "Madame Nazimova—A Comparison," by Anna McClure Sholl; and "Apprenticeship in Letters," by Rene Bache. Some exceptionally good verses and the usual department of humor "Walnuts and Wine," complete the number.

"For the last few years pharmacsts and physicians working hand in hand, have set themselves to change some of their mutual errors and mistakes of the past. It lies not in the mouth of the physician to reproach the pharmacist nor in the mouth of the pharmacist to reproach the physician. We have erred mutually, we have erred together, nd we are determined to redeem ourselves together. The mere trade in patent medicines, in frauds and fakes, he deceitsof all kinds, need not concern us. There are crimes outside of the ranks of medicine and outside of the ranks of pharmacy and we are not starting off on a general reform expedition. There are other organizations and other agencies for that purpose, but the movement to make the drugs—whether the product of the manufacturing houses or the product of the individual pharmacist—which are dispensed over the counter, upon our prescriptions, what they purport to be is one in which you and we have a common interest, and in which our patients have the greatest interest of all. I recognize and you recognize—we must recognize—that in the general progress of science and the general advance of discovery, and the general progress of the arts of manufacturing and preparation of crude pharmaceutics, there is abundant room for large manufacturing houses which devote themselves to specialties of various kinds.

### MANUFACTURING PHARMACY.

For example, how can the individual pharmacist undertake to prepare and supply he group of animal extracts and serums, which now have a large part in the therapeutics of today? And so even with various galenicals, alkaloids and the like. There are many things which the retail pharmacist cannot do as well as that establishment which possesses the proper facilities and which is thoroughly organized to do well on a large scale what can only be done on a small scale. We all recognize that, and the American Medical Association has taken steps, individual physicians have taken steps, to place themseives in proper relation with the great manufacturing houses, which are a credit to American Pharmacy and to American business. We went to have the most cordial relations with them, so that these firms may be encouraged to prepare and offer to us for the benefit of our patients the best and purest and most definite pharmaceutical products. And yet, after all, there is a place, and there must be a place always for the individual pharmacist—the retail druggist, call him by whatever name you please; for the individual who practices as a scientific man the profession of pharmacy."

Some of the indications for Sanmetto are: Vesical irritation and atony; enuresis due to atony; incontinence of urine in children, due to a weak bladder; dribbling of the urine in the aged, not due to paralysis or growths; urine expelled upon exertion, as coughing; cystitis, catarrhal dischargs from bladder or genitalia of male or female; seminal emissions; prostatitis, enlarged prostate and pre-senility.

---

### AVOIDANCE OF ANESTHETIC AND SURGICAL SHOCK

The chance of anesthetic and surgical shock may be greatly lessened or avoided by proper preparation of the patient, whereby there is secured restoration of normal activety of the liver, in particular, kidneys and skin, together with normal elimination by the bowels and correction of acid toxemia. Inadequate preparation of the patient is responsible for more deaths than operations themselves, for as a rule patients requiring operation are not up to the normal standard of health. It is hence a great risk to submit them to operation without sufficient preparation. Sulpho-Lythin, which is a true hepatic stimulent, replaces calomel in all cases where that drug is indicated, and is indispensable in the preparation of patients for operation and during and after treatment of convalescense, because it may be taken continuously without injury. Its judicious use in preparing patients for operation assists in obviating both anesthetic and surgical shock by restoring functional activety of the liver and excretory organs, and counteracting acid toxemia.—Chicago Clinic, May 1906.

---

### INDOOR AIR IN WINTER.

A Wll-known writer says, "Those who have experience will tell you that some of the most inspiring houre of their lives have been spent in active movement in the open air. Their best work is really done there and then; afterward, in their offices and studios, they do but formulate or carry into effect what has already been conceived and determined afoot in the free air."

How important it must be in offices, studios and especially in the homes where we spend the greater part of our time, that the air be pure with the life supporting oxygen where the work can be done under the best conditions.

The presence of watery vapor in normal air varies with the temperature. When air can take no more moisture, it is said to be saturated, and air best adapted for health contains 50 to 75 per cent of saturation.

As soon as the weather becomes sufficiently cold to require heat in buildings, there is a lack of moisture in indoor air, even with the best possible ventilation, no matter what sort of heating apparatus is used, from the old log chimney fire, to the modern steam heat.

Special provision must be made for this. A furnace has a water-box which allows moist air to be carried through the pipes, but stoves and radiators give only the driest sort of heat to the rooms, which is not best suited for breathing and is not so comfortable for the body.

Moist air is aso more readily heated than air having a very low degree of saturation, and to quickly get the benefit of the fuel used, is a desirable point of houe heating.

Keep the furnace water-box filled with clean water, and place on stoves and under all radiators, a good sized receptacle of water; it is surprising how readily the water evaporates.

Pour a little of Piatt's Chlorides into these vessels, and the warm air will not only be healthfully moist, but this odorless disinfectant will keep it free from noxious gases. The ounce of prevention is worth a pound of cure, always.

---

FROM L. ch. BOISLINIERE, M.D.
LATE PROFESSOR OF OBSTETRICE, ST. LOUIS MEDICAL COLLEGE.
BEST UTERINE TONIC AND ANTISPASMODIC.

Dioviburnia is the best Uterine Tonic and Antispasmodic, relieving the pains of Dysmenorrhea and regulator of the Uterine functions. I cheerfully give this recommendation of Dioviburnia.

FROM W. L. CAHAGAN, M.D.,
CORONER OF HAMILTON COUNTY, CHATTANOOGA,
BETTER RESULTS THAN FROM ANY COMBINATION.

I must say that Neurosine has given better results and more universal satisfaction than combination ever used by me. I have tried it in many nervous affections and in Epilepsy of long standing. In some cases it is a specific,, in others a therapeutic agent of great value.

---

HOME-MADE BUTTERMILK.

It is not within the power of every household to have an abundance of that refreshing and healthful summer (also winter) drink—buttermilk. To the present time no one knew of any source of buttermilk except from the butter-maker; but now-a days the butter-maker does his work so well that the butter-milk is entirely deprived of the delicious grains of fat which add so much to its food qualities as well as to taste. True butter-milk, made direct from fresh rich milk, within a few hours, of the finest flavor and taste, nutritious and more excellent than the article as originally known, can now be prepared in any kitchen. This is done by taking a quart of fresh, rich milk, adding a pinch of salt and about a half pint of hot water to raise the temperature to body heat, and lastly adding a tablet which contains a pure culture of lactic bacteria. Place all in a pitcher, cover with a napkin, and let stand for twenty to twenty-four hours at the ordinary temperature, and there is your perfect butter-milk. The tablets are made by Parke, Davis and Co., the pharmaceutical and chemical manufacturers of Detroit, Michigan, and are called "Lactone" or butter-milk tablets.

On the farm, in the process of buttermaking the cream is allowed to sour spontaneously and is then churned. The souring is the lactic acid fermentation caused by lactic acid becteria or ferments. The difference between the old and'ne wprocess is one of method and not result. In the old,, the lactic fermentation is waited for and expected to occur spontaneously, with disappointment sometimes. In the new, the ferment in pure culture is directly planted in the milk, and the desired fermentation is secured without fail. In Bible days, spontaneous fermentation of dough was depended upon to leaven or lighten bread, and failure frequently attended the process, the dough putrefying instead of fermenting, and was then lost. Finally, man learned to add yeast to the dough and not to depend upon spontaneous processes, with the result of always securing the right fermentation and making a better and more nutritious bread. This new buttermilk process is a like improvement.—*Monthly Bulletin Indiana State Board of Health, June* 1907.

A.J. Ronginsky believes that in sterility the male plays an important part. It may result from masturbation, excessive small penis, or varicocele; finally it is necesary to examine several specimens of seamen and ascertain that the spermatozoa are absolutely normal. In the woman there may be general muscular relaxation, lack of function on account of age, or excess of sexual excitability. Of organic changes we may enumerate almost all the gynecological troubles. The principal are congenital lack of development of some part of the genital tract, leucorrhea, stenosis, gonorrheal tube affections. The author discards infantile uterus as a cause. Appropriate remedies are suggested for each of these conditions. In conclusion he exhorts the physician to educate the public to the knowledge of the baneful effects of gonorrhea on the fertility of the sexual organs.—Medical Record, June 22, 1907.

### SUBSTITUTION.

The following letter from Fairchild and Foster indicates the extent to which substitution is being practiced. Satisfactory results certainly cannot be attained unless the drugs indicated are embodied in our prescriptions. The Doctor, not the Druggist is censured for bad results.

"Dear Sir:— We received a request from the Ways and Means Committee of the N. A. R. D. to subscribe to the fund for the entertainment of the members of the N. A. R. D. at its forthcoming Convention in the City of Chicago. In view of previous experience, it occured to us to take steps to ascertain to what extent that "community of interest" expressly implied and naturally anticipated, did actually prevail. We therefore caused prescriptions, in which Essence of Pepsine, Fairchild's, was plainly specified, to be sent to the following mentioned drug stores.

I. M. Light,
143 East 35th. Street.,
Herman Lieberman,
52 So. Halsted St.,
J. S. Link,
649 West 21st. St.,
C. P. Girten,
7100 Harvard Ave.,
H. J. Holthoefer,
N.W. cor. State & 32nd. Sts.,
John J. Boehm,
748 So. Halsted St.,
William W. Klore,
2354 State St.,
William P. Knoche,
N.E. cor. 61st. & Halsted Sts.,
Moyen Bros.,
1595 Milwaukee Ave., cor. Armitage.

Each vial so dispensed was upon receipt immediately sealed in its original wrapper and delivered to us, with complete data in every detail from the writing to the dispensing and sealing of the prescription. Upon examination the fluid dispensed by each of these druggists was found to be a substitute—some preparation not made by us and not Fairchild's Essence.

These substitutes were unlike in composition; they were of varying degrees of inferiority *and some practically worthless* for the purpose for which Fairchild's Essence of Pepsine is esteemed and employed.

There is one significant fact that should also be mentioned, and that is that the price at which the substitutes were sold is that charged the patient by pharmacists who dispense Fairchild's Essence of Pepsine when it is ordered.

We therefore bring these facts to your attention, again stating our intention to fight afainst substitution and for a "square deal."

Very truly yours,

Fairchild Bros and Foster.

